# Expression and Significance of AQP3 in Cutaneous Lesions

**DOI:** 10.1155/2021/7866471

**Published:** 2021-10-26

**Authors:** Dongfeng Niu, Yanhua Bai, Qian Yao, Wei Hou, Lixin Zhou, Xiaozheng Huang, Chen Zhao

**Affiliations:** Key Laboratory of Carcinogenesis and Translational Research (Ministry of Education), Department of Pathology, Peking University Cancer Hospital & Institute, Beijing, China

## Abstract

Aquaporin 3 (AQP3) is the membrane channel of water and involved in fluid homeostasis. The aim of this study was to reveal the expression and significance of AQP3 in cutaneous lesions. We analyzed AQP3 mRNA levels using RT-PCR in 311 cutaneous lesions and confirmed AQP3 expression in these lesions by immunohistochemistry. AQP3 mRNA was detected in normal epidermis, seborrheic keratosis, solar keratosis, Bowen's disease, squamous cell carcinoma, eccrine poroma, apocrine carcinoma, and sebaceoma; however, AQP3 mRNA was absent in basal cell carcinoma, nevocellular nevus, or malignant melanoma. By immunohistochemistry, diffuse AQP3 expression was seen in all keratotic lesions including seborrheic keratosis, verruca vulgaris, molluscum contagiosum, solar keratosis, Bowen's disease, and squamous cell carcinoma. Diffuse AQP3 expression was also present in all extramammary Paget's disease. No AQP3 staining was obtained in basal cell carcinoma. Positive AQP3 staining was seen in sweat gland tumors including hidradenoma, eccrine poroma, and apocrine carcinoma. Among sebaceous tumors, AQP3 expressed diffusely in all sebaceous hyperplasia and sebaceous adenoma, but not in sebaceous carcinomas. Only focal AQP3 staining was seen in nevocellular nevus and no AQP3 staining in melanoma. Our findings indicate the function of AQP3 maintained in most skin tumors. AQP3 may be used for differential diagnosis in skin tumors.

## 1. Introduction

Aquaporin 3 (AQP3) is a water-transporting aquaglyceroporin and plays a significant role in physiologic functions of reabsorption and secretion in the kidney [1, 2], epidermis [3, 4], pancreas [5], prostate [6], etc. The AQP3 protein expression was observed in some normal tissue such as pituitary cells, salivary gland, thymic epithelium, bronchial epithelial cells and pulmonary alveolar epithelium, pancreatic islet, and squamous epithelium of the esophagus, uterine cervix, skin [7], etc. Furthermore, AQP3 has been detected in malignancy of several organs including the colon [8], ovary [9], breast [10], pancreas [11], and prostate [12]. Expression of AQP3 in these tumors was reported to correlate with tumorigenesis, invasion, metastasis, and proliferation [8–13]. In a previous study, we found that AQP3 protein was widely distributed in neoplastic tissues including skin squamous cell carcinomas by immunohistochemical staining [7].

Skin cancer is one of the most common human malignancies with increasing incidence worldwide [14–16]. Skin cancer is mainly divided into two large groups: cutaneous melanoma and nonmelanoma skin tumor. Malignant melanoma is one of the most aggressive human cancers and causes 75% mortality in all skin cancer in the United States [17]. Nonmelanoma skin tumor accounts for nearly 95% of cutaneous neoplasms, and the most common lesions include basal cell carcinoma, squamous carcinoma, and sebaceous carcinoma [15]. Frequent exposure to ultraviolet radiation or sunlight acts as the major factor for both groups of skin cancer [14, 17]. With the development of clinical examination, such as dermoscopy and in vivo reflectance confocal microscopy, the diagnosis and differential diagnosis of skin cancer have become more precise.

In a recent study [7], we found that AQP3 protein was immunohistochemically diffusely positive in the cytoplasmic membrane of normal cutaneous cells including squamous epithelium, sudoriferous gland, sebaceous gland, and apocrine gland but not in melanocytes. We also found a high positive expression of AQP3 in skin squamous carcinomas. However, limited information on AQP3 expression in other skin tumors is available. In this study, we investigated the expression and significance of AQP3 in a large series of skin lesions, using RT-PCR and immunohistochemistry.

## 2. Materials and Methods

### 2.1. Case Selection

Our study was approved by the local research ethics committee of Peking University Cancer Hospital & Institute. We included 311 surgically resected skin lesions from the routine surgical pathology file of Peking University Cancer Hospital & Institute, including 74 benign nonneoplastic lesions (14 seborrheic keratoses, 16 verruca vulgaris, 13 sebaceous hyperplasias, 5 molluscum contagiosum, and 26 nevocellular nevi), 40 benign skin tumors (7 hidradenomas, 7 eccrine poromas, 16 sebaceomas, and 10 sebaceous adenomas), and 197 premalignant lesion and malignant tumors (24 solar keratoses, 26 Bowen's diseases, 43 squamous cell carcinomas, 32 basal cell carcinomas, 16 extramammary Paget' disease, 9 sebaceous carcinomas, 19 apocrine carcinomas, and 28 malignant melanomas). The cases are summarized in Table 1. The diagnosis of all these cases was confirmed by 2 pathologists (DN and YB). The blocks for the study also included normal tissues adjacent to the lesions.

### 2.2. RNA Extraction and RT-PCR

Total RNA was isolated from various formalin-fixed and paraffin-embedded skin lesion tissues with Recover ALL™ Total Nucleic Acid Isolation Kit (Applied Biosystems, USA). We designed the specific PCR primers targeted for AQP3 and PGK1 (as an internal control) as shown in Table 2. HotstarTaq DNA polymerase kit (Qiagen, USA) was performed for amplification. PCR conditions were described previously [11].

### 2.3. Immunohistochemical Staining

The immunohistochemical staining was performed as described previously [7]. The rabbit polyclonal anti-AQP3 antibody was obtained from Sigma (HPA014924, Sigma, USA, 1 : 4000 dilution). The expression of AQP 3 was evaluated as negative (<1% cells positive for AQP3), focal (1 to 9% of positive cells), intermediate (10 to 50% of positive cells), and diffuse (51% or more positive cells). Staining was individually evaluated by two observers (DN and YB) blinded to all clinicopathological information.

## 3. Results

### 3.1. AQP3 mRNA Expression in the Skin Lesions

**Figure 1:**
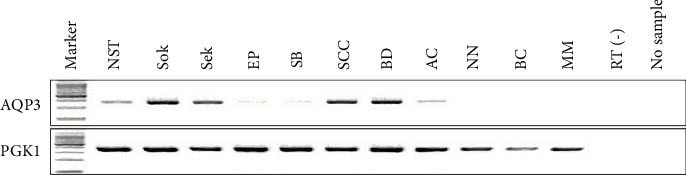
AQP3 mRNA level in skin normal tissue and lesions. RT-PCR showed AQP3 mRNA in normal squamous tissue (NST), solar keratosis (SoK), seborrheic keratosis (SbK), eccrine poroma (EP), sebaceoma (SB), squamous cell carcinoma (SCC), Bowen's disease (BD), and apocrine carcinoma (AC), but AQP3 mRNA was absent in nevocellular nevus (NN), basal cell carcinoma (BC), and malignant melanoma (MM).

We first detected and analyzed the expression pattern of the AQP3 mRNA in the normal squamous epithelium and skin lesions by RT-PCR. As shown in Figure 1, the bands of AQP3 were seen in normal skin tissues (NST), solar keratosis (SoK), seborrheic keratosis (SbK), eccrine poroma (EP), sebaceoma (SB), squamous cell carcinoma (SCC), Bowen's disease (BD), and apocrine carcinoma (AC) but not expressed in nevocellular nevus (NN), basal cell carcinoma (BC), and malignant melanoma (MM). These results were in accordance with our immunohistochemical observation.

### 3.2. AQP3 Expression in Normal Control Tissue

**Figure 2:**
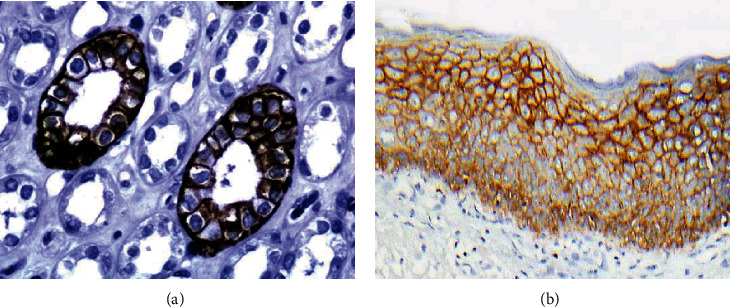
Immunohistochemical staining (immunoperoxidase staining in detail) of AQP3 in normal kidney and skin tissue, kidney tissue as positive control. Immunoreactivity of AQP3 is shown in the cytoplasmic membrane of collecting ducts and squamous cell (magnification: 400x).

We used the sections from normal kidney tissue as a positive control. The expression of AQP3 was specifically localized in the cytoplasmic membrane of the collecting duct cells of the kidney (Figure 2(a)) and squamous cells of the skin (Figure 2(b)) as previously described [7].

### 3.3. AQP3 Expression in Benign Skin Lesions and Tumors

**Figure 3:**
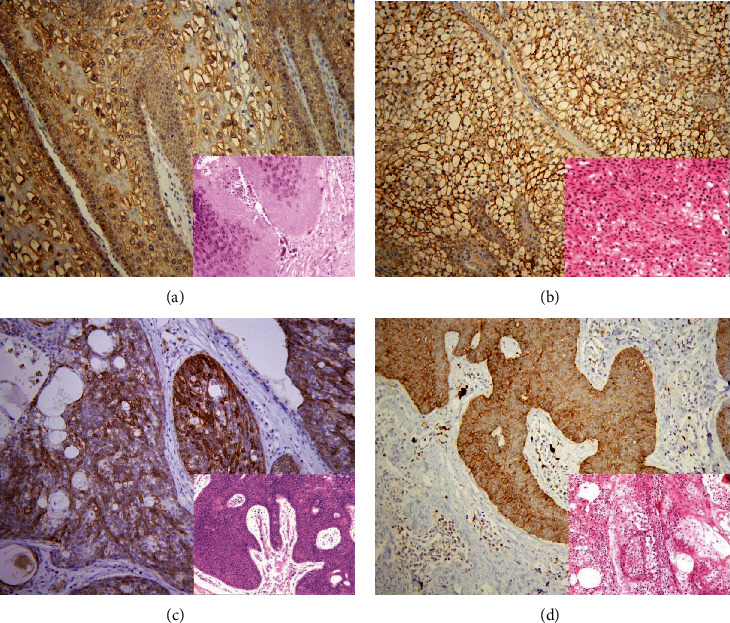
Expression of AQP3 in benign skin lesions and tumors. Distinctive AQP3 immunoreactivity is identified in molluscum contagiosum (a), hidradenoma (b), eccrine poroma (c), and sebaceoma (d) with corresponding figure of HE staining on the lower right-hand corner (magnification: (a, b) 400x; (c, d) 200x).

The immunohistochemical staining of AQP3 in skin lesions is listed in Table 1. Among the nonneoplastic skin lesions, all cases of seborrheic keratosis (*n* = 14), verruca vulgaris (*n* = 16), molluscum contagiosum (*n* = 5), and sebaceous hyperplasia (*n* = 13) showed diffuse staining for AQP3 (Figures 3(a)–3(d)). Among the 26 nevocellular nevi, 8 showed focal AQP3 staining and the remaining 18 were negative. Among the benign skin tumors, positive AQP3 staining was seen in all 7 hidradenomas (all diffuse), 6/7 eccrine poromas (all diffuse), all 16 sebaceomas (4/16 focal, 4/16 intermediate, and 8/16 diffuse), and all 10 sebaceous adenomas (all diffuse).

### 3.4. AQP3 Expression in the Premalignant Lesion and Malignant Skin Tumors

**Figure 4:**
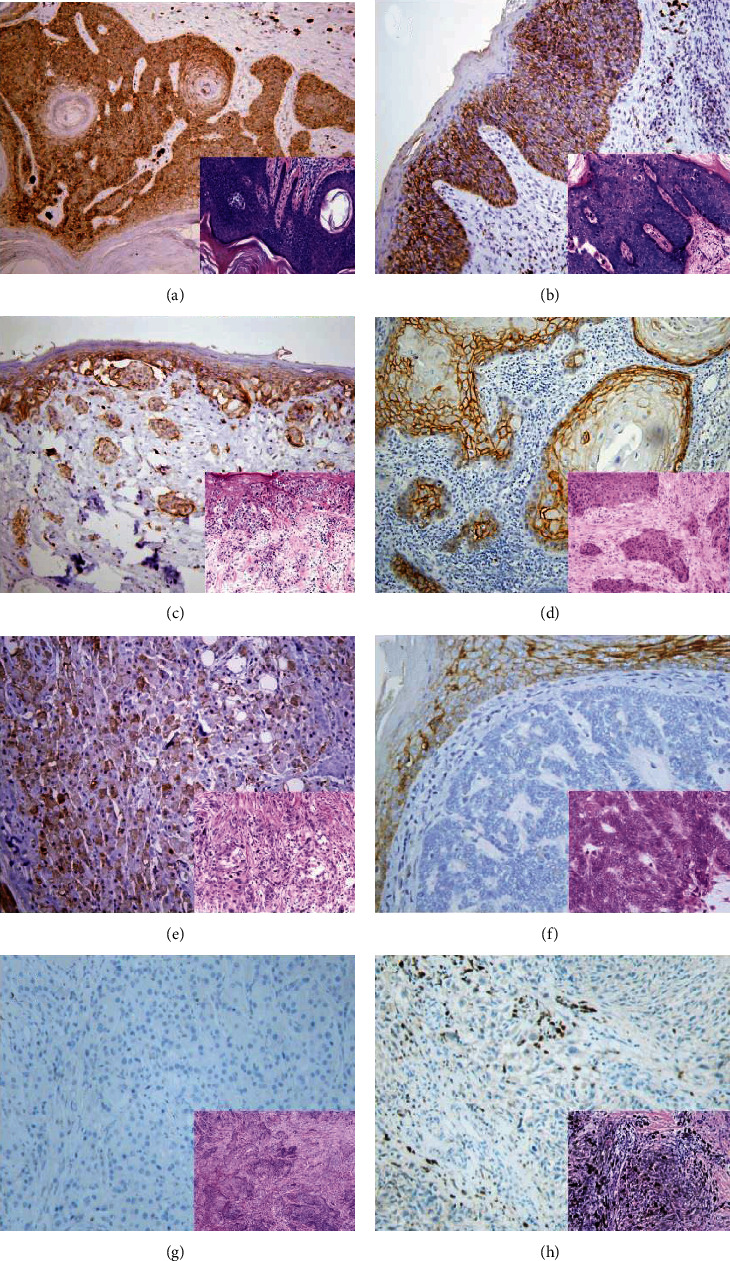
AQP3 expression in malignant skin tumors. Diffuse cytoplasmic membrane staining of AQP3 is identified in solar keratoses (a), Bowen's disease (b), Paget's disease (c), squamous cell carcinoma (d), and apocrine carcinoma (e), while negative AQP3 staining is observed in basal cell carcinomas (f), sebaceous carcinomas (g), and malignant melanomas (h) with corresponding figure of HE staining on the lower right-hand corner (magnification: 200x).

The immunohistochemical results of AQP3 staining in premalignant solar keratoses and malignant skin tumors are summarized in Table 1. All 24 solar keratoses (Figure 4(a)) and 16 extramammary Paget's diseases (Figure 4(c)) showed diffuse AQP3 staining. Positive AQP3 staining was seen in 26 Bowen's diseases (Figure 4(b)) (2 focal, 24 diffuse), 43 squamous cell carcinomas (Figure 4(d)) (3 focal, 7 intermediate, and 33 diffuse), and 19 apocrine carcinomas (Figure 4(e)) (3 intermediate, 16 diffuse). All 32 basal cell carcinomas (Figure 4(f)), 9 sebaceous carcinomas (Figure 4(g)), and 28 malignant melanomas (Figure 4(h)) were negative for AQP3 staining.

## 4. Discussion

Aquaporin3 (AQP3), one of the aquaglyceroporins, is responsible for transporting water and glycerol and maintaining fluid homeostasis in normal tissues [18–20]. In our study, we found that most skin tumors, except basal cell carcinoma, sebaceous carcinoma, and melanoma, showed positive AQP3 staining (diffuse in most positive tumors). No AQP3 staining was seen in basal cell carcinomas, sebaceous carcinomas, and malignant melanomas. In our previous study [7], we showed that AQP3 staining was present in normal epidermal cells and sweat gland cells. Our findings support the view that the water homeostasis regulated by AQP3 was well maintained during carcinogenesis in most of the skin tumors except basal cell carcinoma, sebaceous carcinoma, and malignant melanoma.

No expression of AQP3 in basal cell carcinoma and sebaceous carcinoma might help to elucidate their histogenesis and/or pathogenesis. Basal cell carcinoma usually arises from the lowermost layer of the epidermis and less commonly from the outer root sheath of the pilosebaceous unit. Basal cell carcinoma cells share many common features with follicular epithelium such as hair bulbs, follicular bulges, and follicular matrix cells [21]. In our previous study, we did not observe AQP3 expression in hair follicles [7]. No expression of AQP3 in basal cell carcinoma further confirms the hair follicle origin of basal cell carcinoma. Sebaceous carcinoma shows no expression of AQP3 whereas benign sebaceous lesions including sebaceous hyperplasia, sebaceous adenoma, and sebaceoma all show retained expression of AQP3, suggesting that loss of AQP3 may contribute to the carcinogenesis of sebaceous carcinoma.

The absent expression of AQP3 in basal cell carcinoma and sebaceous carcinoma also has some diagnostic value. In clinical practice, sometimes, it is a big challenge to distinguish basal cell carcinoma from basaloid squamous cell carcinoma or metaplastic basal cell carcinoma from poorly differentiated squamous cell carcinoma. AQP3 immunohistochemical staining is helpful in this situation. Since both basal cell carcinoma and sebaceous carcinoma show negative AQP3 staining, AQP3 is not useful to distinguish basal cell carcinoma with sebaceous differentiation from sebaceous carcinoma. As for sebaceous tumors, which consist of sebaceoma, sebaceous adenoma, and sebaceous carcinoma, their differential diagnosis depends mainly on histological architectural, cytological features, and mitotic rate. Only immunoreactivity of p53 has been reported to act as a useful marker to distinguish between benign and malignant sebaceous carcinomas [22, 23]. In our study, we found the diffuse positivity pattern of AQP3 expressed in all sebaceous hyperplasias and sebaceous adenomas, but not in sebaceous carcinomas. Therefore, AQP3 immunohistochemical staining may have some value for distinguishing benign sebaceous tumors from malignant ones.

It is not surprising that malignant melanoma shows no expression of AQP3 as normal melanocytes within the epidermis do not show AQP3 expression as demonstrated by our prior study [7].

## 5. Conclusion

In summary, our results showed that AQP3 is expressed in most skin tumors except basal cell carcinoma, sebaceous carcinoma, and malignant melanoma, reflecting the biological characteristic of skin tumors. AQP3 has some diagnostic utility in differential diagnosis of skin tumors.

## Figures and Tables

**Table 1:** Summary of immunohistochemistry for AQP3 in skin lesions.

		*N*	Absent	Focal	Intermediate	Diffuse
Nonneoplastic lesions	Seborrheic keratosis	14	0	0	0	14
	Verruca vulgaris	16	0	0	0	16
	Sebaceous hyperplasia	13	0	0	0	13
	Molluscum contagiosum	5	0	0	0	5
	Nevocellular nevus	26	18	8	0	0

Benign tumors	Hidradenoma	7	0	0	0	7
	Eccrine poroma	7	1	0	0	6
	Sebaceoma	16	0	4	4	8
	Sebaceous adenoma	10	0	0	0	10

Premalignant lesion and malignant tumors	Solar keratosis	24	0	0	0	24
	Bowen's disease	26	0	2	0	24
	Squamous cell carcinoma	43	0	3	7	33
	Basal cell carcinoma	32	32	0	0	0
	Paget's disease	16	0	0	0	16
	Sebaceous carcinoma	9	9	0	0	0
	Apocrine carcinoma	19	0	0	3	16
	Malignant melanoma	28	28	0	0	0

Absent, negative; mild, focal (1 to 9% of cells); moderate, intermediate (10 to 50%); diffuse (more than 50%).

**Table 2:** Primer pairs used in RT-PCR.

Target	Gene accession	Primer sequence	AT (°C)	Product size (bp)
AQP 3	NM_004925	F: 5′-GACAGAAGGAGCTGGTGTCC-3′	58	199
		R: 5′-AGAGTGACAGCAAAGCCAAAG-3′		

PGK1	NM_000291	F: 5′-GCTGACAAGTTTGATGAGAAT-3′	58	359
		R: 5′-AGGACTTTACCTTCCAGGAGC-3′		

## Data Availability

The data used to support the findings of this study are available from the corresponding author upon request.
